# Increased hepatic FDG uptake on PET/CT in hepatic sinusoidal obstructive syndrome

**DOI:** 10.18632/oncotarget.11816

**Published:** 2016-09-01

**Authors:** Honsoul Kim, Song-Ee Baek, Jieun Moon, Yun Ho Roh, Narae Lee, Arthur Cho

**Affiliations:** ^1^ Department of Radiology, Yonsei University College of Medicine, Seoul, Republic of Korea; ^2^ Research Institute of Radiological Science, Yonsei University College of Medicine, Seoul, Republic of Korea; ^3^ Biostatistics Collaboration Unit, Yonsei University College of Medicine, Seoul, Republic of Korea; ^4^ Department of Nuclear Medicine, Yonsei University College of Medicine, Seoul, Republic of Korea

**Keywords:** syndrome, sinusoidal obstruction syndrome

## Abstract

**Purpose:**

Imaging features of sinusoidal obstruction syndrome (SOS), an increasingly common drawback of chemotherapy, were evaluated via 18F-fluorodeoxyglucose (FDG) positron emission tomography computed tomography (PET/CT).

**Experimental Design:**

This retrospective study was approved by our Institutional Review Board, with a waiver of informed consent. FDG PET/CT studies of 35 patients (male, 24; female, 11; median age, 53.2 years) obtained between January, 2005 and December, 2012 were analyzed before and after systemic chemotherapy. Diagnosis of SOS was based on histologic (*n*=13) or gadoxetic acid-enhanced MRI (*n*=22) findings. On PET/CT images, ROIs drawn on non-tumorous liver generated mean standardized uptake value (SUV_liver_). Total lesion glycolysis of liver (TLG_liver_) was calculated as: SUV_liver_ × CT-derived hepatic volume. Paired t-test was applied to compare changes before and after SOS.

**Results:**

Mean (±standard error [SE]) values of hepatic volume (baseline, 1307.7±46.2 cm^3^; SOS, 1395.4±41.3 cm^3^; *p*=0.004), SUV_liver_ (baseline, 2.08±0.06; SOS, 2.27±0.07; *p*=0.02), and TLG_liver_ (baseline, 2697.5±114.5; SOS, 3170.2±134.2; *p*=0.001) significantly increased with development of SOS. In contrast, mean SUV_aorta_ was unchanged (baseline, 1.53±0.04; SOS, 1.50±0.04; *p*=0.52).

**Conclusions:**

Hepatic FDG uptake on PET/CT intensified after onset of SOS and thus may be an inappropriate reference in this setting, potentially skewing chemotherapeutic responses gauged by lesion-to-liver SUV ratio.

## INTRODUCTION

Recent advances in chemotherapeutic regimens, surgical techniques, and imaging studies have markedly improved treatment outcomes in various types of cancer [1-4]. Regimens incorporating oxaliplatin and cisplatin have significantly improved treatment response rates, enabling surgical resection of initially unresectable liver metastases in carefully selected patients [5-9]. However, these newer agents are not without side effects and often induce hepatic sinusoidal obstruction syndrome (SOS) [10]. As their usage increases, a commensurate upsurge in SOS may well be anticipated.

Although SOS is not life-threatening to the majority of sufferers, its detection has important clinical ramifications in certain patients. Hematopoietic stem cell transplant recipients are particularly vulnerable to SOS, with reported mortality of ∼80-90% [11, 12]. Furthermore, patients with hepatic SOS are more prone to bleeding and therefore are at greater risk of intra- and post-operative complications [13-15].

Radiologic findings indicative of SOS are well known, reflecting the morphology that is manifested in liver [16]. Heterogeneous hepatic parenchymal enhancement during the portal phase of CT has been described in SOS and may be diffuse, peripheral, or multifocal in distribution [17]. Reticular hepatic hypointensity on the hepatobiliary phase of gadoxetic acid-enhanced MRI is also a highly specific feature of SOS [18-20]. As such, we assumed that these SOS-related morphologic and/or functional changes would similarly appear on 18F-fluorodeoxyglucose positron emission tomography/computed tomography (FDG PET/CT) imaging.

FDG PET/CT enables non-invasive whole-body imaging of glucose metabolism and has been increasingly utilized to predict responses to systemic chemotherapy in patients with cancer. Nevertheless, only one case report of SOS-related PET/CT imaging attributes is currently found in the literature [21]. Moreover, the liver-to-lesion SUV ratio (SUV_lesion_/SUV_liver_) determinable by PET/CT is a frequent means of assessing tumor treatment response. Thus any change in SUV_liver_ during development of SOS is of considerable importance.

The purpose of this study was to investigate hepatic FDG PET/CT findings in the context of SOS, comparing PET/CT scans performed before and after its development.

## RESULTS

### SOS-related changes in metabolism, serum analytes, and platelet count

**Figure 1:**
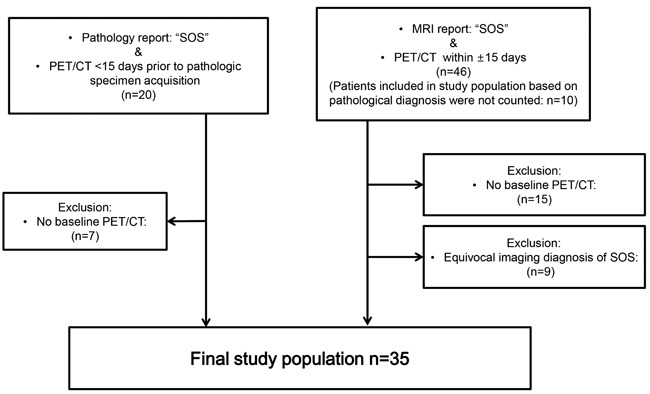
Eligibility criteria of study population

The basic demographic and clinical characteristics of patients with SOS are provided in Figure 1 and Table 1. Changes in metabolic and biochemical indices of patients with SOS and control group are summarized in Table 2 and supplementary Table 1, respectively.

**Table 1:** Demographic and clinical characteristics of patient population

Characteristics	Data (*n*=35)
Age, median (range)	53.2 (34.7–78.9)
Gender (M:F)	24 (69%): 11 (31%)
Primary malignancy (n,%)	35 (100%)
Colon/rectum	17 (49%)
AGC/duodenum	12 (34%)
Pancreas/AOV	3 (9%)
GIST	1 (3%)
Lymphoma	1 (3%)
Ovary	1 (3%)
Chemotherapy (n)	35 (100%)
Oxaliplatin-based (Eloxatin/Oxalitin/Pleoxtin)	28 (80%) (19/7/2)
Cisplatin-based	6 (17%)
CHOP	1 (3%)
Interval between chemotherapy and second PET/CT (months)	5.6 ± 3.0

AGC: advanced gastric cancer; GIST: gastrointestinal stromal tumor; AOV: ampulla of Vater

**Table 2:** Comparison of biochemical indices in patients developing SOS

	Baseline	SOS	Mean change	95% confidence	*p*-value
Weight (kg)	61.7±1.5	59.6±1.7	-2.0±0.8	-3.6–-0.4	0.014*
Bilirubin (mg/dL)	0.47±0.03	0.76±0.06	0.29±0.05	0.2–0.4	<0.001*
AST (IU/L)	24.3±3.7	50.9±11.7	26.6±11.9	2.3–50.8	0.032*
ALT (IU/L)	21.9±3.6	44.3±12.4	22.3±12.6	3.2–47.8	0.09
Cr (mg/dL)	0.83±0.03	0.79±0.03	0.03±0.03	-0.09–0.02	0.22
CEA (ng/mL)	11.6±3.4	5.0±1.1	-6.6±3.1	-13.0–-0.2	0.044*
ALK (IU/L)	66.5±3.7	90.6±6.1	24.1±5.2	15.5–34.8	<0.001*
Platelet count (×103/uL)	258.4±14.9	161.1±16.7	-97.3±23.5	-145.1–-49.5	<0.001*
Glucose	93.0±9.1	94.3±13.1	1.3±13.8	-3.7–6.3	0.595

* Statistically significant (paired t-test)

Indices expressed as mean±SE

Following onset of SOS, mean patient weight significantly declined (baseline, 61.7±1.5 Kg; SOS, 59.6±1.7 Kg; *p* = 0.014), whereas mean serum levels of bilirubin (baseline, 0.47±0.03 mg/dL; SOS, 0.76±0.06 mg/dL; *p* < 0.001), aspartate transaminase (baseline, 24.3±3.7 IU/L; SOS; 50.9±11.7 IU/L; *p* = 0.032), and alkaline phosphatase (baseline, 66.5±3.7 IU/L; SOS, 90.6±6.1 IU/L; *p* < 0.001) increased significantly. Other indices, namely alanine transaminase (*p* = 0.09), and creatinine (*p* = 0.22), similarly tended to increase, but not to a statistically significant extent. A significant reduction in mean platelet count (baseline, 258.4±14.9 10^3^/uL; SOS, 161.1±16.7 10^3^/uL; *p* < 0.001) was also observed.

### SOS-related changes in organ volume and FDG PET/CT parameters

Changes in hepatic and splenic volumes and in PET/CT indices of patients with SOS are listed in Table 3. Mean sizes of liver (baseline, 1307.7±46.2 cm^3^; SOS, 1395.4±41.3 cm^3^; mean change, 87.7±28.7 cm^3^; % change, 8.5%; *p* = 0.004) and spleen (baseline, 164.6±10.9 cm^3^; SOS; 240.5±15.8 cm^3^; mean change, 76.5±12. cm^3^; % change, 51.3%, *p* = 0.001) increased significantly following onset of SOS (Table 3). In contrast, no significant change in the hepatic and splenic volume was observed in the control group (Supplementary Table 2).

**Table 3:** Comparison of PET/CT parameters in patients developing SOS

	Baseline	SOS	Mean change	95% confidence	*p*-value	% change
Hepatic volume (cm^3^)	1307.7±46.2	1395.4±41.3	87.7±28.7	29.4–146.0	0.004*	8.5±2.6
Splenic volume (cm^3^)	164.6±10.9	240.5±15.8	76.5±12.0	52.0–101.0	0.001*	51.3±7.5
SUV_liver_	2.08±0.06	2.27±0.07	0.19±0.08	0.04–0.35	0.02*	12.2±4.4
SUV_aorta_	1.53±0.04	1.50±0.04	-0.03±0.05	-0.13–0.07	0.52	-0.04±3.5
SUV_ratio_	1.36±0.02	1.53±0.03	0.16±0.04	0.09–0.24	<0.001*	12.8±2.7
TLG_liver_	2697.5±114.5	3170.2±134.22	472.7±124.5	219.7–725.6	0.001*	21.0±4.7
SUV_spleen_	1.66±0.07	1.79±0.06	0.13±0.07	0.01–0.28	0.074	12.0±4.5
TLG_spleen_	268.9±18.5	424.6±29.3	155.7±25.9	102.9–208.5	0.001*	68.7±10.5

Parameters expressed as mean±SE

* Statistically significant (paired t-test)

TLG_liver_: total lesion glycolysis of liver; SUV_ratio_ (Liver-to-blood ratio) = SUV_liver_ / SUV_aorta_

**Figure 2:**
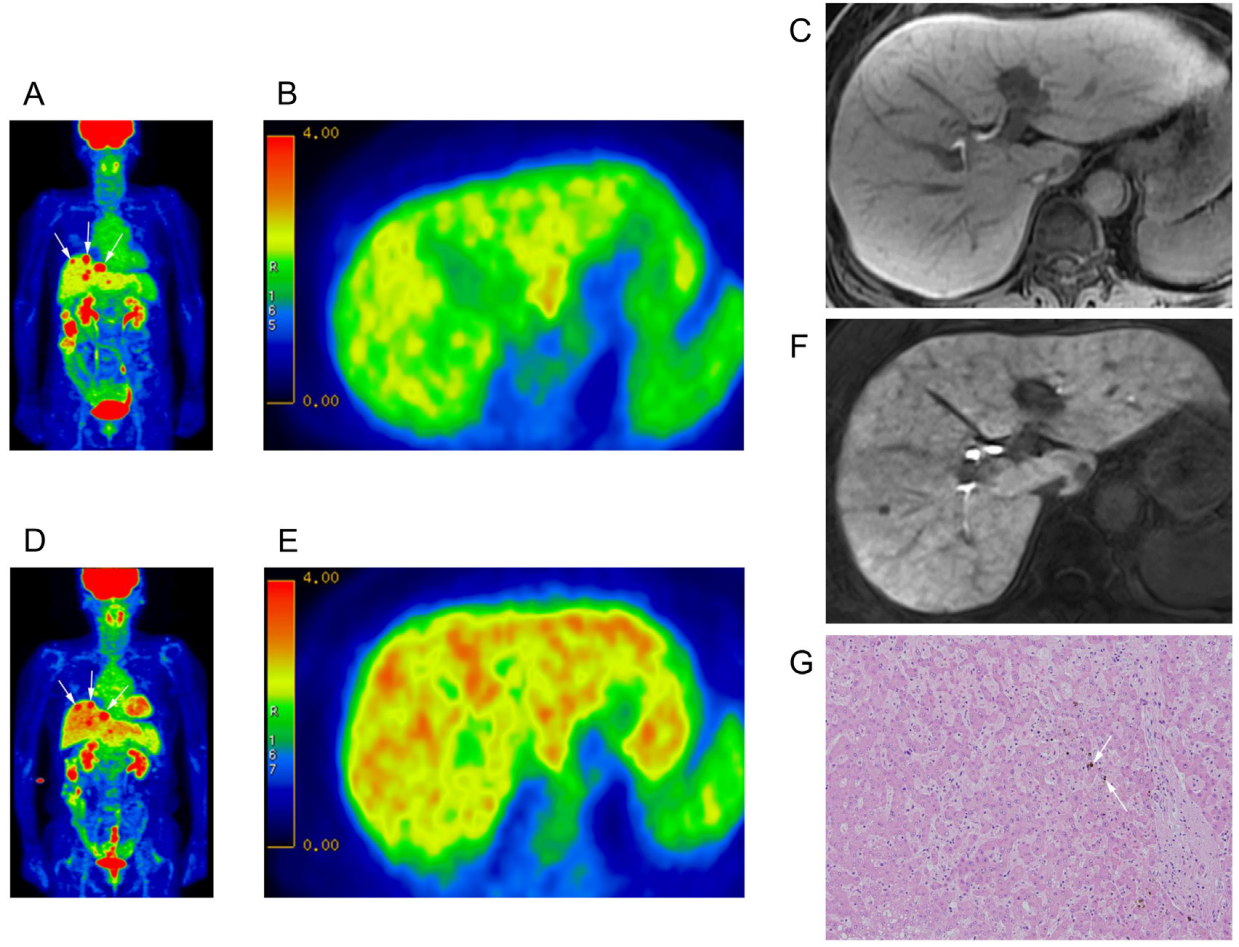
Intensified FDG uptake in patient developing SOS **A.**-**C.** Baseline 18F-FDG PET/CT: **A.** Maximum intensity projection (MIP) image of 74 year-old woman with ascending colon cancer and multiple hepatic metastases (arrows); **B.** axial PET images (mean SUV, 2.3; hepatic volume, 1134.5 mm^3^; TLG, 2609.35); and **C.** hepatobiliary phase image of gadoxetic acid-enhanced MRI (prior to oxaliplatin chemotherapy). **D.**-**F.** 18F-FDG PET/CT after FOLFOX regimen (8 cycles): **D.** Maximum intensity projection (MIP) image showing diffuse increase in hepatic FDG uptake. Multiple hepatic metastases are observed (arrows); **E.** axial PET image (mean SUV, 2.6; hepatic volume, 1205.3 mm^3^; TLG, 3133.78); and **F.** hepatobiliary phase of gadoxetic acid-enhanced MRI. **G.** Microscopic view of surgically resected tumor-free liver with morphologic changes of sinusoidal obstructive syndrome such as diffuse sinusoidal dilatation and intracytoplasmic cholestasis (arrows), (H&;E stain ×100).

All metabolic indices indicated a significant upsurge in hepatic FDG uptake (relative to baseline FDG PET/CT) following onset of SOS, with a significant rise in mean SUV_liver_ (baseline, 2.08±0.06; SOS, 2.27±0.07; % change, 12.2%; *p* = 0.02) and in the blood pool-adjusted SUV_ratio_ (baseline, 1.36±0.02; SOS, 1.53±0.03; % change, 12.8%; *p* < 0.001) (Figure 2). Mean TLG_liver_ also increased significantly (baseline, 2697.5±114.5; SOS, 3170.1±134.2; increment, 472.7±124.5; % change, 21.0%; *p* = 0.001), signaling a rise in metabolic glycolysis. Mean SUV_aorta_ remained unchanged (baseline, 1.53±0.04; SOS, 1.50±0.04; % change, -0.04%; *p* = 0.52) (Table 3). Meanwhile, no significant alteration in the SUV_liver_, SUV_aorta_, SUV_ratio_ and TLG_liver_ between the first and second PET/CT was observed in the control group (Supplementary Table 2).

### Severity of SOS and FDG PET/CT

As set forth by Chao [22], SOS in our cohort was graded as mild (*n* = 23), moderate (*n* = 9), or severe (*n* = 3), using the highest rank warranted by at least one of four determinants; and patients were stratified as mild (*n* = 23) or moderate/severe (*n* = 12) for correlation with imaging indices. Compared with the mild SOS subset, TLG_liver_ trended higher in patients with moderate/severe SOS (2995.7±144.0 *vs* 3504.7±259.7; *p* = 0.104), falling short of statistical significance, whereas mean splenic volume (212.6±18.1 *vs* 291.6±24.7; *p* = 0.017) and mean TLG_spleen_ (363.6±28.5 *vs* 536.4±51.8; *p* = 0.003) were significantly higher. Other metabolic or imaging indices did not differ significantly by severity of SOS (Table 4).

**Table 4:** Comparison of PET/CT indices by severity of SOS

	Mild SOS (*n* = 23)	Moderate/severe SOS (*n* = 12)	*p*-value
Hepatic volume (cm^3^)	1368.6±55.4	1446.9±56.8	0.376
SUV_liver_	2.2±0.1	2.4±0.2	0.178
SUV_aorta_	1.5±0.04	1.5±0.1	0.52
SUV_ratio_	1.5±0.03	1.6±0.1	0.234
TLG_liver_	2995.7±144.0	3504.7±259.7	0.104
Age	55.6±2.53	55.5±3.27	0.975
Splenic volume (cm^3^)	212.6±18.1	291.6±24.7	0.017*
SUV_spleen_	1.8±0.1	1.8±0.1	0.534
TLG_spleen_	363.6±28.5	536.4±51.8	0.003*

* Statistically significant

Indices expressed as mean±SE

## DISCUSSION

In this study, we have demonstrated that hepatic FDG uptake on PET/CT increases after onset of SOS. To the best of our knowledge, only one published case report has similarly delineated this relationship. Known risk factors of SOS, such as adjuvant or neo-adjuvant chemotherapy, hematopoietic stem cell transplantation, and liver transplantation [13, 22, 23], should be sought when interpreting PET/CT images. Prior oxaliplatin-based chemotherapy is a critical factor, given that the risk of subsequent SOS is considerable (18.9-79%) [24-26]. CT and/or MRI imaging conducted in parallel may provide further diagnostic support. Hallmark features of SOS include heterogeneous parenchymal enhancement on contrast-enhanced dynamic liver studies and/or reticular hypointensity during hepatobiliary phase of a gadoxetic acid-enhanced MRI [17-20]. Hepatic or splenic enlargement, readily detectable in imaging studies, is also characteristic of SOS (Table 3). A suspected shift in hepatic FDG uptake may be exposed through careful comparison of sequential PET/CT studies (current *vs* baseline) in terms of SUV_liver_.

Because PET/CT is frequently utilized to gauge chemotherapeutic response, hepatic FDG uptake is an important clinical parameter, serving in general as a standard reference. Hence, FDG uptake by various tumors may be compared within or among individuals by calculating lesion-to-liver SUV ratio (SUV_lesion_/SUV_liver_). This strategy, based on an authenticated concept that SUV_liver_ is quite stable over time [27], is also integral to the PET Response Criteria in Solid Tumors (PERCIST) 1.0 protocol. The latter guides therapeutic response determinations, utilizing liver to normalize SUL (i.e., lean body mass SUV).

Our findings instead indicate that hepatic FDG uptake can be affected by the development of SOS, and as a result sometimes may change rather than remain constant. In our cohort, approximately 10% increase in SUV_liver_ was observed on PET/CT (relative to baseline) after onset of SOS (Table 3). Consequently, the lesion-to-liver SUV ratio (SUV_lesion_/SUV_liver_) will decline by default, and may have more or less influence on the interpretation of chemotherapy response assessment. Furthermore, SOS is a dynamic process, subject to aggravation, improvement, or resolution [22, 26]. Therefore, SUV_liver_ may actually vacillate (as would lesion-to-liver SUV ratio and even SUL) in this setting, rather than simply increasing, according to phase of disease. However, we found no statistically significant difference in SUV_liver_ determinations when stratifying patients by severity of SOS (Table 4).

On the other hand, SUV_aorta_ remained consistent, regardless of whether or not SOS developed (Table 3, 4). According to PERSIST (1.0), if the liver is diseased and not suitable to serve as a reference, the blood pool activity in descending aorta is an acceptable alternative [28]. In view of this finding, we advise all pertinent specialists to be cognizant of SOS and its clinical presentation and urge that descending aorta (rather than liver) serve as diagnostic reference.

The mechanism for intensification of hepatic FDG uptake with onset of SOS is unclear. Focal or diffuse FDG uptake by tissues (organ or lesion) is typically attributed to heightened blood flow or glucose metabolism. Decreased washout and stasis of radiotracer are alternative explanations. Toxic injuries to endothelium have been implicated in SOS, which is histologically characterized by sinusoidal dilatation with peliosis, nodular regenerative hyperplasia, and fibrosis [29]. We speculate that microcirculatory disturbances due to endothelial cell injury and stasis of blood in peliosis may produce hepatic congestion, thereby passively increasing the blood-pool FDG tracer activity. In the sole case report to date, which formally documented a correlation between FDG uptake and SOS [21], trapping of FDG within dilated sinusoids was offered as explanation. Inflammation seemed less likely, considering its conspicuous absence from liver biopsy [21].

This investigation has several acknowledged limitations. Although histologic confirmation of SOS was not routinely achieved, we contend that gadoxetic acid-enhanced MRI is a modality with high specificity for SOS and is a reliable mode of diagnosis, based on published evidence. Our imaging protocol was also fraught with inconsistency (e.g., intervals between baseline and follow-up PET/CT), as an inherent retrospective constraint. In addition, we have assumed that accumulated FDG in congested sinusoids is the basis for intensified FDG uptake in patients with SOS. To evaluate this hypothesis, a dynamic FDG PET/CT protocol is needed, well beyond the scope of this study. Future efforts should explore this method in assessing or grading the severity of SOS. As a final note, we were unable to validate our hypothesis that a rise in SUV_liver_ with onset of SOS may artifactually skew the lesion-to-liver SUV ratio, causing misinterpretation of treatment response. Subsequent research should address this issue.

In conclusion, we have found that SOS is associated with a rise in mean SUVliver on PET/CT, relative to baseline status. Clinical awareness of this phenomenon is paramount, given that chemotherapy predisposes to SOS. The reduced lesion-to-liver SUV ratio (SUV_lesion_/SUV_liver_) that results may create a false impression of therapeutic efficacy.

## MATERIALS AND METHODS

### Patient selection

The study protocol was approved by our Institutional Review Board, with a waiver of informed consent. This was a retrospective study for which all data were kept anonymous. Utilizing the facility's electronic clinical database, patients undergoing FDG PET/CT between January, 2005 and December, 2012 qualified as potential candidates. Inclusion criteria were as follows: 1) recipients of systemic cancer chemotherapy, 2) diagnosis of hepatic SOS, based on either histopathology of surgical specimens (*n* = 13) or gadoxetic acid-enhanced MRI (*n* = 22) findings, and 3) two FDG PET/CT studies at minimum, done at baseline (prior to chemotherapy) and within 15 days of diagnosing SOS (Figure 1). Any diagnosis of SOS arrived at solely by gadoxetic acid-enhanced MRI was reviewed by two radiologists (H.K. and S-E.B.), accruing 4 and 7 years of experience in liver imaging, respectively. A consensus was reached on whether imaging characteristics of SOS were present, namely normal appearing hepatic parenchyma on baseline dynamic contrast-enhanced CT and MRI (if available) and unequivocal diffuse and heterogeneous hepatic parenchymal signal hypointensity on hepatobiliary phase image of gadoxetic acid-enhanced MRI in the aftermath of systemic chemotherapy [18-20].

Ultimately, 35 patients were recruited for study, each diagnosed with SOS and having the requisite FDG PET/CT studies at baseline and after diagnosis of SOS. The following clinical parameters (obtained within 3 days of FDG PET/CT) were recorded to gauge severity of SOS: weight, bilirubin, aspartate transaminase, alanine transaminase, creatinine, carcinoembryonic antigen, alkaline phosphatase, and platelet count. Patients were then categorized as mild, moderate, or severe SOS, as suggested by Chao [22].

### Patient demographics

A total of 35 patients (median age, 53.2 years; range, 34.7-78.9 years) met our study criteria, including 24 men (median age, 55.1 years; range, 42.6-78.9 years) and 11 women (median age, 50.5 years; range, 34.7-74.9 years) whose treated cancers varied in origin (gastrointestinal tract, 29; pancreas, 3; gastrointestinal stromal tumor [GIST], 1; lymphoma, 1; ovary, 1) (Table 1). In the vast majority of patients (28/35, 80.0%), SOS developed after oxaliplatin-based chemotherapy. Six patients received cisplatin, and only one was given a regimen of CHOP (cyclophosphamide, doxorubicin, vincristine, and prednisone) (Table 1). Duration of treatment ranged from 65-330 days (mean, 152.3±70.5 days). The interval between baseline and second PET/CT ranged from 3.0-57.1 months (mean, 11.5±11.8 months). The interval between starting chemotherapy and second PET/CT was on average 5.6 months ± 3.0 months.

In parallel, we defined a control group of 35 individuals (24 males, 11 females; median age, 52.9 years; range, 34.4-78.9 years). These patients did not have any history of malignancy and underwent PET/CT at least twice for health examination. The interval between first and second PET/CT was 22.3 months ± 13.3 months.

### Gadoxetic acid-enhanced MRI protocol

In 22 patients, diagnosis of SOS relied on gadoxetic acid-enhanced MRI views generated by 3-T (Magnetom Tim Trio; Siemens Medical Solutions, Erlangen, Germany) or 1.5-T (Interna Achieva; Philips Healthcare, Best, The Netherlands) imaging unit. Routine in-phase and opposed-phase T1- and T2-weighted images were obtained. The contrast-enhanced dynamic study protocol at our facility consisted of a breath-hold transverse 3D GRE (TR/TE, 2.5/0.9 msec; flip angle, 13°; FOV, 38 cm; matrix, 320 × 224; section thickness, 2 mm; no gap for 3T unit or TR/vTE, 4.4/2.1 msec; flip angle, 15°; FOV, 38 cm; matrix, 256 × 256; section thickness, 2 mm; no gap for 1.5T unit).

To determine the scan delay for arterial phase imaging, a 1-mL test bolus injection technique was used. Contrast-enhanced MRI was performed using a breath-hold 3D-GRE sequence following intravenous (IV) bolus administration of gadoxetic acid (0.025 mmol/kg body at 2 mL/s) and a 20-mL saline flush. Portal venous and transitional phase images were obtained ∼30-40 seconds after acquisition of preceding images, done 20-35 s (arterial phase), 60-70 s (portal venous phase), 100-120 s and 150-180 s (transitional phase) from time of IV contrast injection. All images were oriented in transverse plane. Hepatobiliary phase images were acquired 15-20 min after gadoxetic acid injection in identical manner. Imaging parameters were individualized as needed.

### PET/CT protocol and imaging analysis

All patients underwent routine FDG PET/CT scans (Discovery 600 PET/CT; GE Healthcare, Milwaukee, WI, USA), with fasting for at least 6 hours and confirmed peripheral blood glucose levels ≤140 mg/dl before FDG injection. FDG (∼5.5 MBq/kg) was administered IV 1 hour before image acquisition. After initial low-dose CT (Discovery 600: 30mA, 130 kVp), standard PET imaging was performed from neck to proximal thighs (acquisition time, 3 min/bed) in 3D mode. Image reconstruction was by ordered subset expectation maximization (2 iterations, 20 subsets).

Studies were displayed on a GE AW 4.0 workstation (GE Healthcare, Milwaukee, WI, USA) for review by a nuclear medicine specialist (A.C.) with 10 years of experience. Region of interest (ROI) was drawn in triplicate on non-tumorous hepatic parenchyma. SUVs were normalized to body weight. Mean SUV (SUV_liver_) was recorded and total lesion glycolysis of the liver (TLG_liver_) was calculated as SUV_liver_ × CT-derived hepatic volume (cm^3^). Mean SUV of aorta (SUV_aorta_) was similarly obtained, drawing three 1-cm ROIs on abdominal aorta and recording the average. Liver-to-blood ratio (SUV_ratio_) was calculated as the ratio between SUV_liver_ divided by SUV_aorta_ (SUV_liver_ / SUV_aorta_). These five indices (hepatic volume, SUV_liver_, TLG_liver_, SUV_aorta_, and SUV_ratio_,) were compared at baseline and in conjunction with SOS to evaluate relative change in liver glycolysis.

Hepatic and splenic volumes were determined by a radiologist (H.K.) with 4 years of experience in hepatic imaging. To do so, CT axial images were archived in Digital Imaging and Communications in Medicine (DICOM) format and stored on a GE AW 4.0 workstation (GE Healthcare). An ROI was drawn along the margins of liver or spleen on each slice, and total organ volumes were reached additively, combining ROIs of consecutive images. The following formula was applied to determine % change rate (%) of each parameter: (Parameter_First PET/CT_ - Parameter_Second PET/CT_)/Parameter_First PET/CT_.

### Statistical analysis

Statistical analyses relied on standard software (SPSS v19; SPSS Inc., Chicago, IL, USA). All clinical and imaging data were assessed for normality as continuous variables, using the Kolmogorov-Smirnov test at *p* > 0.05 to fulfill the normality assumption. Parametric analyses were applied to normally distributed variables. Paired *t*-test was utilized to compare changes in clinical and imaging indices before and after development of SOS. Patients were stratified as mild SOS or moderate/severe SOS to improve analytic clarity, and Student's *t*-test was engaged for evaluating differences in imaging indices by degree of SOS. Statistical significance was set at *p* < 0.05.

## SUPPLEMENTARY MATERIALS FIGURES AND TABLES


