# Supplementary material for: Increased hepatic FDG uptake on PET/CT in hepatic sinusoidal obstructive syndrome
Source: Oncotarget. 2016 Sep 1;7(42):69024–31. doi: 10.18632/oncotarget.11816 (PMC5356608; doi:10.18632/oncotarget.11816)
Supplement: Supplementary file 1 [file oncotarget-07-69024-s001.pdf]

# Increased hepatic FDG uptake on PET/CT in hepatic sinusoidal obstructive syndrome

**Supplementary Material**

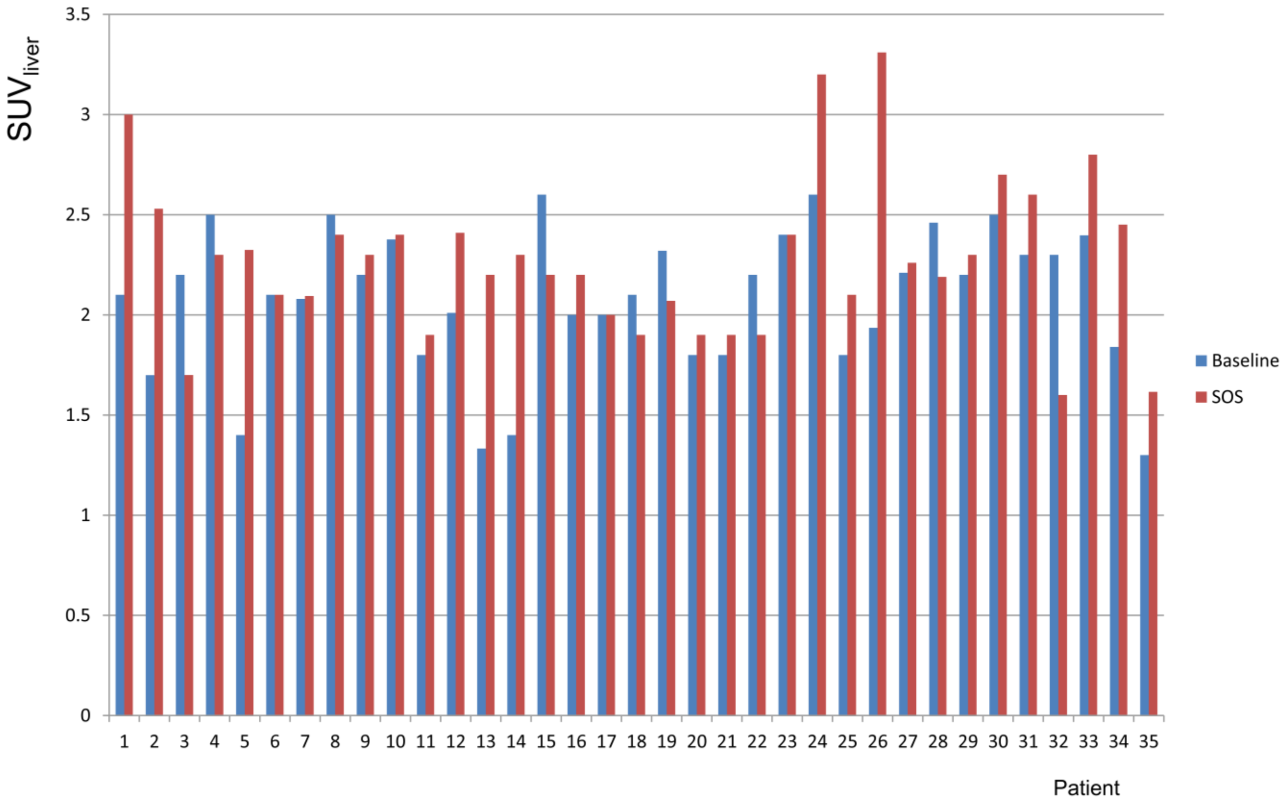


**Supplementary Figure 1. Graph displaying the changes in $SUV_{liver}$ of each patient who developed SOS. Blue lines indicate $SUV_{liver}$ at baseline, and red lines indicate $SUV_{liver}$ after developing SOS.**
**(patient #1-23: mild SOS, Patient #24-35: moderate/severe SOS.)**

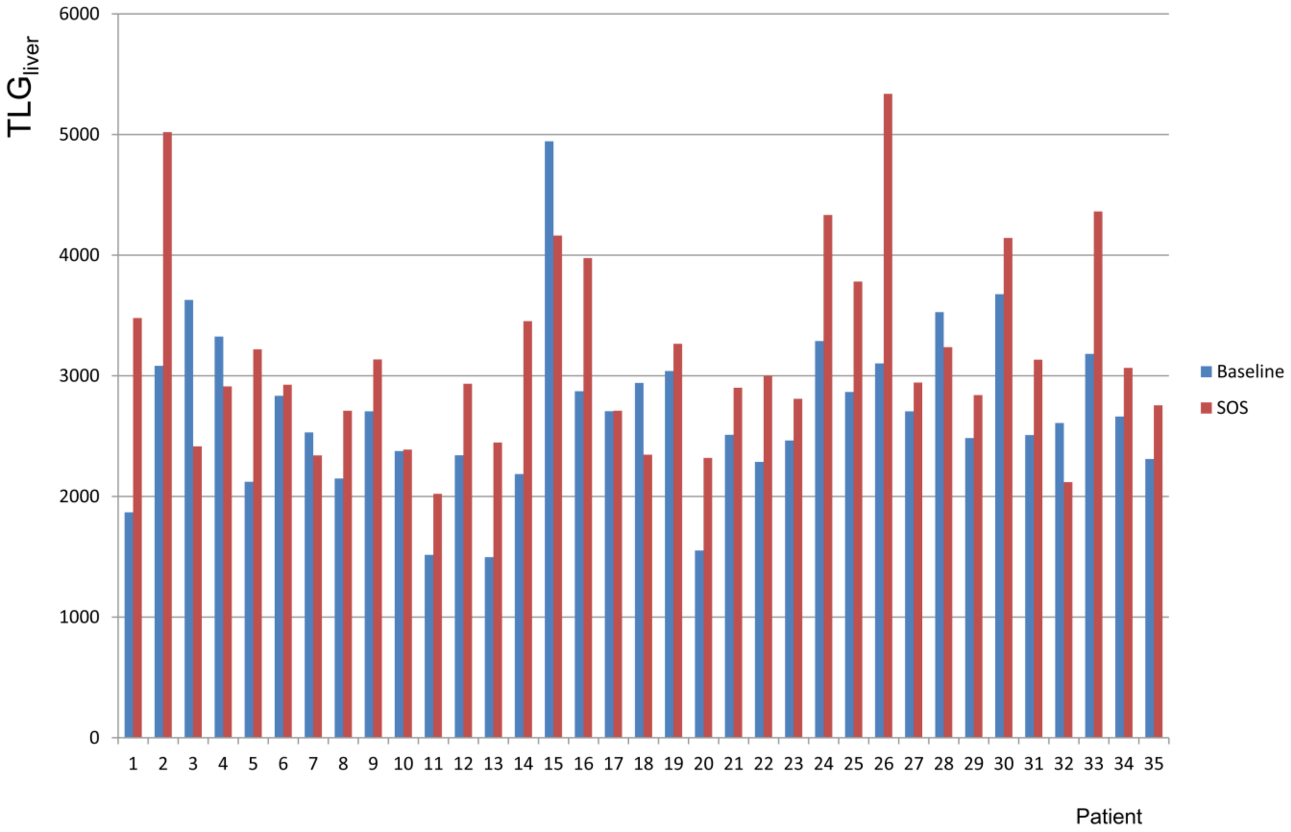


**Supplementary Figure 2. Graph displaying the changes in $SUV_{TLG}$ of each patient who developed SOS. Blue lines indicate $SUV_{TLG}$ at baseline, and red lines indicate $SUV_{TLG}$ after developing SOS.**
**(patient #1-23: mild SOS, Patient #24-35: moderate/severe SOS.)**

**Supplementary Table 1**. Biochemical indices in control group.

| | Baseline (First PET/CT) | Follow up (Second PET/CT) | Mean change | 95% confidence | *p*-value |
|---|---|---|---|---|---|
| Weight (kg) | 67.5±1.9 | 68.5±1.9 | 1.0±0.8 | -0.7–2.7 | 0.235 |
| Bilirubin (mg/dL) | 0.70±0.05 | 0.71±0.04 | 0.01±0.04 | -0.08–0.07 | 0.855 |
| AST (IU/L) | 23.4±1.3 | 23.1±1.6 | -0.3±1.5 | -3.3–2.8 | 0.859 |
| ALT (IU/L) | 28.2±2.8 | 25.5±3.0 | -2.7±2.3 | -7.5–2.0 | 0.246 |
| Cr (mg/dL) | 0.92±0.04 | 0.88±0.04 | -0.04±0.02 | -0.07–-0.004 | 0.03* |
| CEA (ng/mL) | 1.48±0.19 | 1.81±0.25 | 0.32±0.20 | -0.08 – 0.73 | 0.116 |
| ALK (IU/L) | 55.5±2.0 | 49.1±1.7 | -6.3±1.5 | -9.5 – -3.2 | <0.001* |
| Platelet count ($\times 10^3$/uL) | 224.0±8.4 | 218±9.4 | -6.0±6.4 | -19.1–7.0 | 0.351 |

***** Statistically significant (paired t-test)
Indices expressed as mean ± SE

**Supplementary Table 2**. Comparison of PET/CT parameters in control group

| | First PET/CT | Second PET/CT | Mean change | 95% confidence | *p*-value | % change |
|---|---|---|---|---|---|---|
| Hepatic volume ($cm^3$) | 1335.3±49.1 | 1309.1±47.2 | -26.2±25.6 | -78.3–25.9 | 0.313 | -1.3±2.0 |
| $SUV_{liver}$ | 2.24±0.06 | 2.35±0.05 | 0.11±0.06 | -0.01–0.2 | 0.071 | 7.42±3.72 |
| $SUV_{aorta}$ | 1.61±0.04 | 1.62±0.05 | 0.00±0.05 | -0.1–0.1 | 0.950 | 1.26±3.05 |
| $SUV_{ratio}$ | 1.39±0.03 | 1.47±0.03 | 0.08±0.04 | -0.00–0.16 | 0.053 | 7.63±3.82 |
| $TLG_{liver}$ | 2996.8±151.8 | 3088.3±131.7 | 91.5±99.7 | -111.5–294.6 | 0.365 | 6.0±4.3 |

Parameters expressed as mean±SE

$TLG_{liver}$: total lesion glycolysis of liver; $SUV_{ratio}$ (Liver-to-blood ratio) = $SUV_{liver}$ / $SUV_{aorta}$

***** Statistically significant (paired t-test)
